# The VISTA datasets, a combination of inertial sensors and depth cameras data for activity recognition

**DOI:** 10.1038/s41597-022-01324-3

**Published:** 2022-05-18

**Authors:** Laura Fiorini, Federica Gabriella Cornacchia Loizzo, Alessandra Sorrentino, Erika Rovini, Alessandro Di Nuovo, Filippo Cavallo

**Affiliations:** 1 University of Florence, Department of Industrial Engineering, Florence, 56139 Italy; 2 Scuola Superiore Sant’Anna, The BioRobotics Institute, Pontedera, PI 56025 Italy; 3 Sheffield Hallam University, Sheffield Robotics and the Department of Computing, Sheffield, United Kingdom

**Keywords:** Biomedical engineering, Scientific data, Information technology

## Abstract

This paper makes the VISTA database, composed of inertial and visual data, publicly available for gesture and activity recognition. The inertial data were acquired with the SensHand, which can capture the movement of wrist, thumb, index and middle fingers, while the RGB-D visual data were acquired simultaneously from two different points of view, front and side. The VISTA database was acquired in two experimental phases: in the former, the participants have been asked to perform 10 different actions; in the latter, they had to execute five scenes of daily living, which corresponded to a combination of the actions of the selected actions. In both phase, Pepper interacted with participants. The two camera point of views mimic the different point of view of pepper. Overall, the dataset includes 7682 action instances for the training phase and 3361 action instances for the testing phase. It can be seen as a framework for future studies on artificial intelligence techniques for activity recognition, including inertial-only data, visual-only data, or a sensor fusion approach.

## Abstract (summary)

**Table:** 

Measurement(s)	movement of upper and lower limbs
Technology Type(s)	IMU • camera

## Background & Summary

Over the last years, human activity recognition has gained considerable success due to the importance of understanding what a person is performing during normal daily activities. The correct understanding of human-being activities is playing a fundamental role also in the field of human-robot interaction (HRI). Indeed, robots can collaborate with humans and help people accomplish their actions. The recognition of activities can be achieved by exploiting different kinds of sensors, such as inertial or visual ones. However, no single modality sensor can cope with all the situations that occur in the real world; just to give an example, if the person makes two very similar gestures involving the whole body that differ only in the movement of the hand, the camera cannot correctly differentiate the two, while an inertial sensor placed on the hand can; indeed it can capture the fine movement of the hands, while the camera is focused on the recognition of the total body movement (i.e. wide movement). Additionally, the presence of multiple and multimodal sensors can introduce redundancy to the system, thus improving the accuracy of the activity recognition system.

In the literature, many open datasets are available for human activity recognition, as shown in Table 1. In the context of human activity recognition, accelerometers are the most widely used sensors thanks to their low power consumption and their ability to capture body movements meaningfully. One of the datasets which exploits this technology is the FallAllD^1,2^, which consists of a multitude of files collected using three data-loggers worn on the wrist, waist and neck of the participants. In the UniMiB dataset^3^, a Samsung Galaxy Nexus equipped with a Bosh acceleration sensor has been used. The University of Dhaka (DU) Mobility Dataset (MD)^4^ is another publicly available dataset built using a single wrist-mounted wearable sensor. In addition to ADLs, the latter three datasets also contain data related to different types of falls. olor video cameras are also often used in human activity recognition to monitor several human people activities. As an example, the Multiview Cooking Actions dataset (MoCA)^5^ is a bi-modal dataset in which six VICON infrared cameras are used to collect Motion Capture data and video sequences from multiple views of upper body actions in a cooking scenario. Even if this approach is very interesting, it is based on a very complex and cumbersome system such as the VICON, which cannot be transported and easily installed in a domestic environment. Furthermore, only upper limbs data are present in the MoCA dataset. A similar but more convenient system for monitoring people at home is based on RGB-D cameras, which are used in the following works to collect data. The RGBD-HuDaAct^6^ is a human activity video database containing synchronized color-depth video streams of 12 daily living activities. Similarly, the Cornell Activity Dataset^7,8^, that contains CAD-60 and CAD-120, comprises RGB-D video sequences and tracked skeletons of four subjects performing activities which are recorded using the Microsoft Kinect sensor. Another very important dataset is the MSRDailyActivity3D^9^, which has been created by using a Kinect device.

**Table 1:** A comparison of the VISTA dataset with existing benchmarks.

Dataset	Sensorsa	Activitiesb
FallAllD1, 2	**Wide**: accelerometer, gyroscope, magnetometer, barometer on the chest, wrist or waist; **Fine**: -	**Basic Gestures**: several types of fall; walking, sitting down, lying down,..; **ADL**: jogging, clap hands, climbing stairs,..; **Scene**: -
UniMiB dataset3	**Wide**: Samsung Galaxy Nexus smartphone equipped with a Bosh acceleration sensor; Fine: -	**Basic Gestures**: several types of fall; walking, standing up, lying down, sitting down; **ADL**: running, going downstairs, going upstairs, jumping; **Scene**: -
DU-MD Dataset4	**Wide**: single wrist-mounted wearable sensor; **Fine**: -	**Basic Gestures**: several types of falls; walking, sitting, laying, standing; **ADL**: jogging, staircase climbing, staircase down; **Scene**: -
RGBDHuDaAct6	**Wide**: color video camera and depth sensor; **Fine**: -	**Basic Gestures**: get up, sit down, stand up, enter and the room; **ADL**: make a phone call, mop the floor, go to bed, get up, eat meal, drink water, take off the jacket and put on the jacket; **Scene**: -
Cornell Activity Dataset7, 8 (CAD-60)	**Wide**: Microsoft Kinect camera; **Fine**: -	**Basic Gestures**: talking on couch; **ADL**: Rinsing mouth, brushing teeth, wearing contact lenses, talking on the phone, drinking water, opening pill container, cooking (chopping), cooking (stirring), relaxing on couch, writing on whiteboard, working on computer; **Scene**: -
MSR Daily Activity 3D9	**Wide**: Microsoft Kinect camera; **Fine**: -	**Basic Gestures**: sitting still, lying down on sofa, walking, standing up, sitting down; **ADL**: drinking, eating, reading book, calling cell phone, writing on a paper, using laptop, using vacuum cleaner, cheering up, tossing paper, playing games, playing guitar; **Scene**: -
MEX dataset10	**Wide**: Pressure mat, a depth camera and two accelerometers on the wrist and the thigh; **Fine**: -	**Basic Gestures**: -; **ADL**: exercises related to Musculoskeletal Disorders (MSD), i.e. knee-rolling, bridging, pelvic tilt, the clam, repeated extension in lying, prone punches, superman; **Scene**: -
Up-Fall Detection Dataset11	**Wide**: Five IMUs (waist, thigh, wrist, chest, foot), one EEG headset, two cameras, six infrared in grid; **Fine**: -	**Basic Gestures**: different types of fall; walking, standing, sitting, laying; **ADL**: picking up an object, jumping; **Scene**: -
MoCA Dataset5	**Wide**: six VICON infrared cameras (markers on shoulder, elbow, wrist, palm, index and little finger knuckles); **Fine**: -	**Basic Gestures**: -; **ADL**: 20 cooking actions; **Scene**: preparing an omelet, grating cheese, melting ingredients, making a sandwich, preparing a lemonade
VISTA Datasets	**Wide**: two color and depth cameras; **Fine**: one wearable inertial glove SensHand	**Basic Gestures**: walk; **ADL**: drink, eat, brush teeth, use laptop, write on a paper, talk on the phone, sweep, relax, read; **Scene**: having lunch, house cleaning, relax, working, personal hygiene

^a^‘Wide’ and ‘Fine’ indicate the sensors used to recognize the wide and fine movements, respectively.

^b^The actions were clustered into ‘Basic Gesture’, e.g. walking, sitting, lying, ‘ADL’, related to a specific activity of daily living, and ‘Scene’, which includes all the activities

composed by two or more activities without restrictions in the passage from one to the other.

The above mentioned datasets available in the literature have been created by considering only one modality sensor. However, by combining multiple sensors it is possible to create more complete datasets. Some examples comprehend the MEX dataset^10^ and the Up-Fall Detection Dataset^11^. In this context, we propose the VISTA database as a unique compromise for the recognition of activities of daily living between datasets based on visual sensors and those based on the inertial ones. Particularly, the added values of the VISTA database, that make it unique compared to the others, are:

- **Daily Actions and Activities** For VISTA actions selection we referred to the Cornell Activity Dataset (CAD-60) and the MSR Daily Activity 3D Dataset. Ten actions in common between the two datasets were selected, choosing actions similar in pairs. The rationale is to create a dataset composed of very similar pairwise activities for what concerns the wide body movement, but that differs from fine movements of the hand. In this sense, this dataset could be used to test the accuracy of a recognition system in distinguish very similar movements.
- **Presence of ADL scenes** In addition to providing data related to simple gestures or ADLs, our dataset provides data related to 5 ADL scenes, which are “combined” data of single actions. In this way we provide data related to the individual ADLs and data related to the transitions between one action and another.
- **Multimodal data** The VISTA database proposes the combination of RGB-D cameras and inertial sensors positioned on the fingers and on the wrist of the subjects. This allows the creation of a complete and heterogeneous database, in which the cameras give an indication of the wide movement of the limbs during the movement, while the inertial modules placed on the fingers acquire data that allow to recognize the fine movements, which are the ones that differentiate the most similar actions. The comparison with the other datasets on this aspect is better highlighted in the Table 1 in the column’Sensors’.
- **Multiple views** One of the drawbacks of using a vision system is the camera occlusion that can occur if the subject is not positioned frontally to the camera. The VISTA database offers the simultaneous video records from two different perspectives, in front of the camera and laterally, with the purpose to investigate whether the use of a multi-modal approach could improve the accuracy of the recognition when the subject is not in a frontal position, thus mimicking very realistic operative conditions.
- **Human - Robot Interaction** The VISTA database was acquired from the interaction with the Pepper robot. The participants interacted with the robot and performed what it was explaining and requiring.

These features make the dataset usable in different applications: just to name a few, the recognition of actions and scenes, the analysis of the transitions between the actions, he development of customized machine learning algorithms, the comparison of approaches using different sensor modalities. The implications of such research efforts are important in the field of activity recognition, but also in the field of human-robot interaction, since the understanding of the actions and the context can improve the robot perception ability.

## Methods

In this section we highlight the main properties of our data collection. We start describing the design of the study and the setup, including a description of the acquisition system.

### Study design

#### Phase 1: Single action - Training phase

In the literature, there is not a common agreement on the definition of action or activity recognition. Often, these terms are used as synonymous. Atomic actions^12^ are movements of a person describing a certain motion that can be part of more complex activities, whereas gestures are considered as primitive movements of the body. Activity is hierarchically defined as a sequence of actions^13^. The first part of the experimentation consisted of the training phase, in which each participant had to simulate 10 different actions, sequentially. Each action was carried out for one minute and this phase took a total of almost 15 minutes per subject. The complete description of the selected actions is reported in Table 2. It is worth pointing out that these actions have been selected in such way to have pairs of actions very similar between them, i.e. actions whose wide movement can make them seem equal, but that differ for the fine movement and grip of the hand. This makes the pairs of actions equal for the cameras and different for the inertial modules placed on the hand (Brush teeth (BT), Eat with the fork (EF); Drink from a glass (DG), Talk on the phone (TP); Read a book (RB), Relax on the couch (RC);Write on a paper (WP), Use laptop (UL); Sweep with the broom (SB), Walk (WK)).

**Table 2:** Description of actions and of the associated scenes included in the dataset.

PHASE 1 (Training): each action for one minute			PHASE 2 (Testing): each scene for one minute				
Action	Description	Position	HC	HL	PH	WO	RE
**EF**	Take the fork, eat and put it back	Sitting on the chair		X			
**DG**	Take the glass, drink and put it back	Sitting on the chair		X	X		
**BT**	Take the toothbrush, brush teeth and put it back	Sitting on the chair			X		
**UL**	Type on the keyboard with both hands	Sitting on the chair				X	
**WP**	Take a pen and write on a paper	Sitting on the chair				X	
**TP**	Take the phone, talk on it and put it back	Sitting on the chair				X	X
**WK**	Walk forward and backward repeatedly	Standing	X				
**SB**	Take the broom, sweep and put it back at the end	Standing	X				
**RC**	Sit comfortably on the couch and relax	Sitting on the couch					X
**RB**	Take the book, read it and turn pages repeatedly	Sitting on the couch					X

For the actions, ‘EF’ stands for ‘eat with the fork’, ‘DG’ for ‘drink from a glass’, ‘BT’ for ‘brush teeth’, ‘UL’ for ‘use laptop’, ‘WP’ for ‘write on a paper’, ‘TP’ for ‘talk on the phone’, ‘WK’ for ‘walk’, ‘SB’ for ‘sweep with the broom’, ‘RC’ for ‘relax on the couch’ and ‘RB’ for ‘read a book’. For the scenes, ‘HC’ stands for ‘house cleaning’, ‘HL’ for ‘having lunch’, ‘PH’ for ‘personal hygiene’, ‘WO’ for ‘working’ and ‘RE’ for ‘relax’.

#### Phase 2: ADL Scene composition - Testing phase

The second part of the experimentation was called Testing phase. In this phase the user was required to perform the same actions performed in the first phase but simulating 5 scenes of daily living: Having Lunch (HL), Personal Hygiene (PH), House Cleaning (HC), Working (WO) and Relax (RE). Each scene was performed for one minute. In this time frame, the subject had to execute the different actions composing the scene, as reported in Table 2. Before starting the execution, the robot gave indications on which scene to execute and among which actions he could choose. The person was free to choose when to switch from one action to another and for how long to carry each of them out within the minute, having also the possibility to re-perform them. This phase lasted approximately 15 minutes.

#### Data acquisition tools

**Fig. 1:**
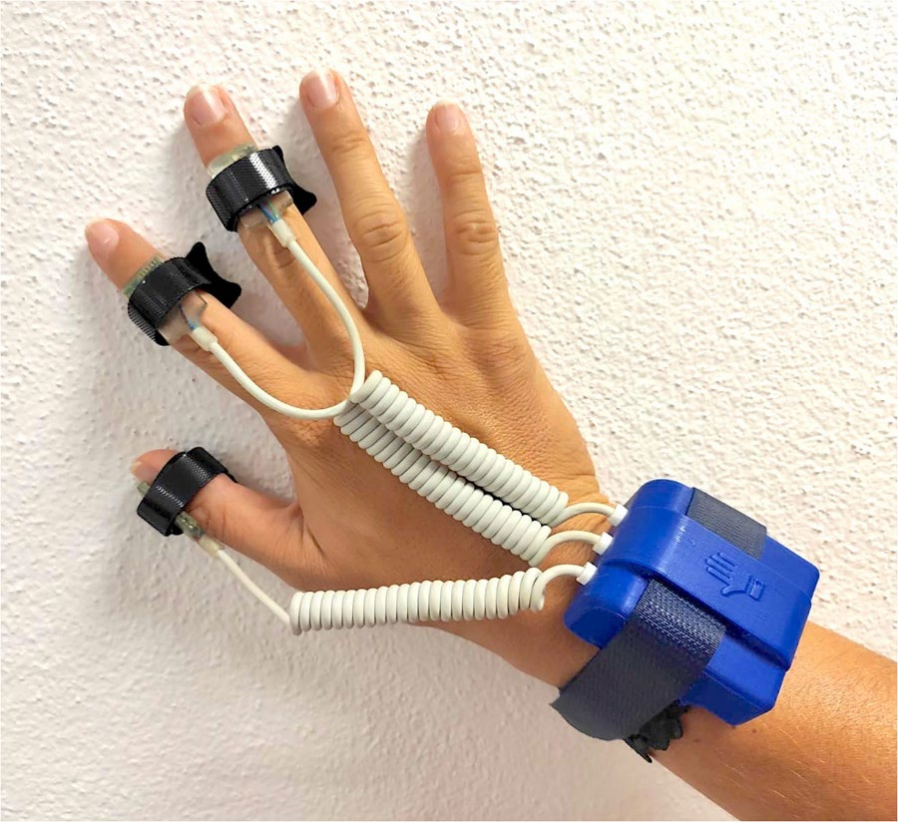
SensHand glove.
- **Pepper robot and RGB-D camera:** Pepper is the world’s first social humanoid robot able to recognize faces and basic human emotions^15^. The robot is characterized by 20 degrees of freedom for natural movements, giving the impression of interacting with a human being. It has touch sensors, speech synthesis, LEDs and microphones for multimodal interactions. An Intel® RealSense™ depth camera has been mounted on Pepper tablet, as showed in Fig. 2, allowing us to obtain colored and depth images, since the latter were not available using only Pepper’s sight. The Intel® RealSense™ depth camera D435i is a stereo tracking solution, which offers RGB images and quality depth for several applications. In order to let Pepper act properly for the experimentation, it was re-programmed through the software Choregraphe: several behaviors, one for each action to be classified, were created and installed on the robot. The depth camera was positioned on the robot’s chest, frontally with respect to the subject. In order to save time, another Intel® RealSense™ depth camera was placed laterally to the subject at the same height, providing an additional point of view. This allowed us to record simultaneously the two video streams during the experimental session without requesting the participant to execute the test twice.

The data were acquired using the following devices:

- **SensHand:** The SensHand is a wearable sensor consisting of four inertial modules placed on the wrist and on the thumb, index and middle finger. In this paper it is used to capture the fine movements of the fingers. Each module is made of a complete 9-axis inertial sensor (6-axis geomagnetic module LSM303DLHC and 3-axis digital gyroscope L3G4200D, STMicroelectronics, Italy) and includes a microcontroller (ARM®-based 32-bit STM32F10RE MCU, STMicroelectronics, Italy) which can acquire, filter and store data at a frequency of 100 Hz^14^. The system is based on a standard Controller Area Network (CAN), where the module placed on the forearm acts as coordinator of the entire system. It collects and transmits data at 100 Hz towards a generic control station through a wireless communication based on the ESD 210 (Parani) Bluetooth serial device. The other modules are positioned on the distal phalanx of the thumb, index and middle finger, as shown in Fig. 1. The device is powered by a small, rechargeable and light-weight Li-Ion battery that ensures autonomy of the system for about four hours.

**Fig. 2:**
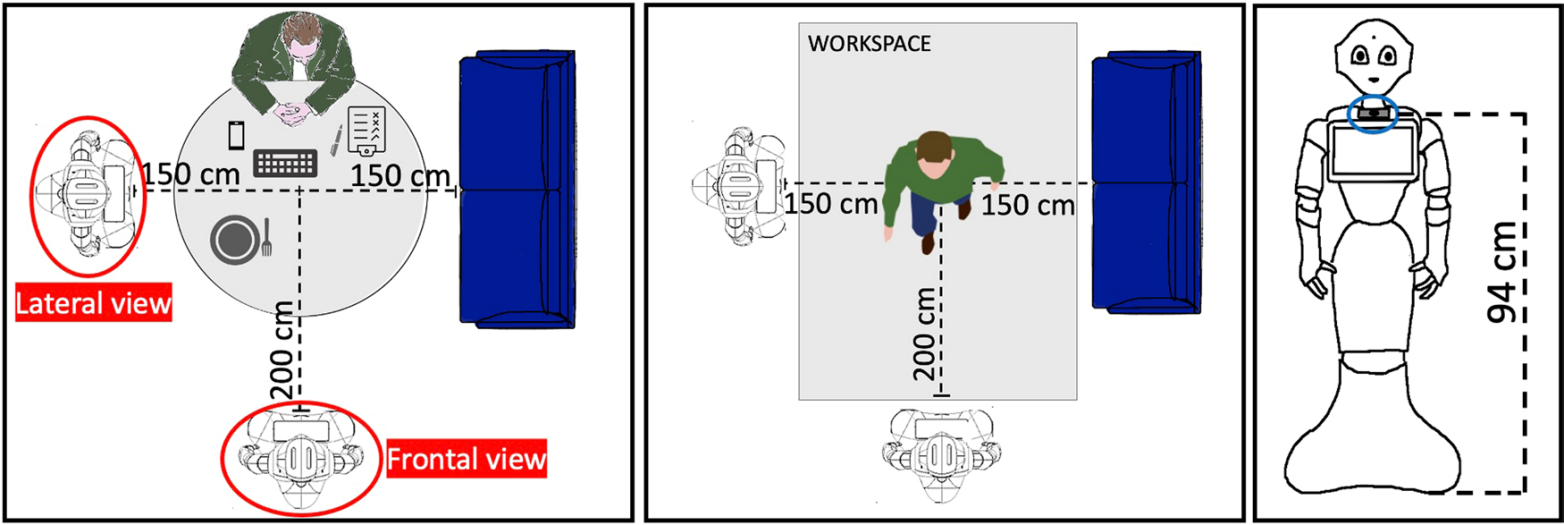
Setup for experimental session.

### Experimental set up

The experimentation was performed in Sheffield (UK), in the Smart Interactive Technology (SIT) research laboratory of the Sheffield Hallam University in a dedicated room. The experimental setup is depicted in Fig. 2.

**Fig. 3:**
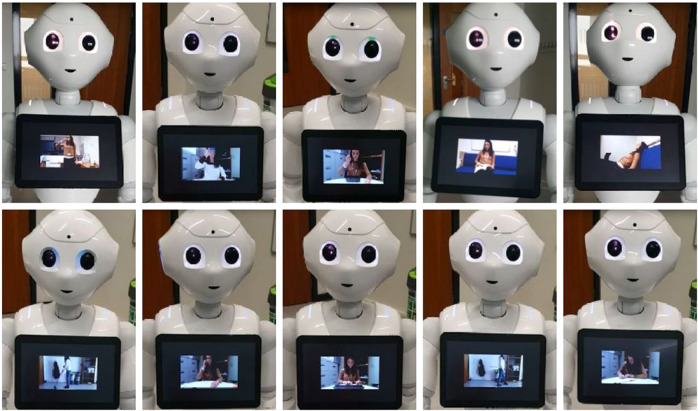
GIFs explaining the movement for the ten activities. Participant has granted the permission to publish these photos.

Before starting the tests, each subject had to wear the SensHand by placing the IMUs on the wrist and on the intermediate phalanges of the thumb, index and middle finger of the dominant hand (as in Fig. 1) and sit in front of the robot. Each participant was requested to perform the training and the test phases. During both phases, Pepper gave instructions to the person on how to perform the actions and the scenes. In order to be sure that the person understood how to perform the action, an explanatory GIF was shown on Pepper’s tablet for the entire duration of the exercise, as showed in Fig. 3. In case of necessity, the user could ask Pepper to repeat the explanation. When the user was ready to start, the robot gave instructions on when to start and when to stop. For the duration of the experimentation, there was no interaction between the robot and the subject for safety reasons; indeed, it was far from the subject during the whole session. Another person was always present in the room to observe the experiment, annotate the data and intervene in case of necessity.

### Participants

Twenty healthy young people were enrolled for the experimental protocol among the staff and the students of the Sheffield Hallam University. They were a cohort of multicultural and right-handed people. Thirteen subjects were female, seven males, and their age ranged from 19 to 44 years old.

### Data synchronization

The data were synchronized through a dedicated web interface. In particular, SensHand sent one data package for each sample. Each package was composed of a series of values from accelerometers, gyroscopes and magnetometers for wrist, thumb, index and middle finger sensors along x, y and z axes. The frequency of acquisition of the wearable glove was 100 Hz. After each acquisition, all information collected was organized in a tabular format in dedicated files. A column containing the timestamps was added for each sample, allowing us to keep track of the time of the signal and synchronise it with the camera’s data during post-processing.

With regard to the acquisition of RGB and depth images from cameras, it was necessary to integrate the two cameras in Robot Operating System (ROS), which comprehends a set of software libraries and tools to develop robot applications. Since two cameras were involved at the same time, it was necessary to start streaming images at the same time. In order to reduce the computational load and speed up the acquisition, compressed images were recorded by the two cameras.

**Fig. 4:**
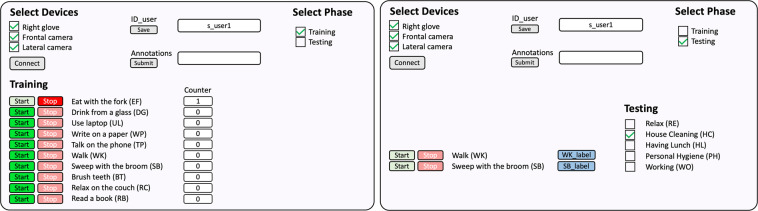
Data collection interface when Training (left) and Testing (right) are selected, respectively.

The creation of this custom interface, showed in Fig. 4, allowed us to customize the saving of information in dedicated rosbag files, that were assembled according to the different phases that constituted the experimentation. As an example, every time the data acquisition started, i.e. every time the button ‘Start’ was pressed, a new .txt file, containing inertial data, and two.bag files, containing images from the two cameras, were created. An artificial counter was added on the interface to keep track of the number of times the’Start’ button was pressed for each action.

### Data annotation

In the VISTA database, the data annotation was performed in real-time by the external observer, who administrated the exercise, through the custom web interface described in the previous paragraph (Fig. 4). During the training phase, the acquisition was unique for each activity and only one action was performed for each session. For this reason, we just saved the name of the corresponding action as name of the training files, e.g. s_user1_t1_bt_1.txt. During the testing phase, we followed a different approach. As it is shown in Fig. 4, when the’Testing’ phase and the desired scene were selected in the interface, the names of the actions concerning that particular scene became visible together with the corresponding button. Each time the person changed activity within the scene, the label button of the new activity was pressed by the external observer. The timestamp and the label were saved in a .txt file. Saved timestamps allowed us to synchronize data.

Table 3 shows the instances’ distribution for the training and testing sequences after annotation. The percentages are computed over the total number of action instances annotated on the training (7682) and testing (3361) set, respectively.

**Table 3:** Activities instances distribution in training and testing sequences.

Activity	Training	Testing
Eat with the fork (EF)	10%	12%
Drink from a glass (DG	10%	15%
Brush teeth (BT)	10%	12%
Use laptop (UL)	10%	7%
Write on a paper (WP)	10%	9%
Talk on the phone (TP)	10%	10%
Walk (WK)	10%	9%
Sweep with the broom (SB)	10%	11%
Relax on the couch (RC)	10%	6%
Read a book (RB)	10%	9%

### Approval of ethics committee

The approval of the ethics committee was obtained from Sheffield Hallam University (UK) Ethical Committee. As a token of gratitude, each participant received an Amazon e-voucher of £10 after completing the experiment. This expense, covered by the HEIF funding allocated for this project, encouraged the participation in the experiment. The participants received an information sheet and they were verbally informed that they had the right to withdraw from the experiment at any time. Participants were asked to indicate age and gender and to fill out the informed consent before the beginning of the procedure in which they consented to the video-recording of the session. To protect information and prevent data from being misused, all the data were stored in an anonymous and secure way. To ensure that the ethical issues were handled properly and that the right to privacy was respected, a codification system in data handling was used. Specific attention was paid to the procedures related to informed consent, the confidentiality of the information, data storage, fair and lawful processing of data. There were no risks or any possible negative consequences for participants: the robot used for the experimental session is a commercial platform, proved safe for HRI, integrated into the standard environment and under constant control by experienced technicians.

## Data Records

Due to privacy restrictions, the VISTA database includes two sections: the *Public dataset* and the *Dataset on request*. Both datasets are stored by the Sheffield Hallam University Research Data Repository (SHURDA)^16^. The *Public dataset* is public and anyone can access and use it. This contains all the inertial data acquired with SensHand, only the depth images acquired by the camera and the spatial coordinates of the skeleton joints extracted from the RGB images. All this information is itself anonymous. The *Dataset on request* is not public and it will be made available upon request and for research purposes only. The *Dataset on request* includes raw RGB and depth images acquired with the camera that couldn’t be anonymised.

**Fig. 5:**
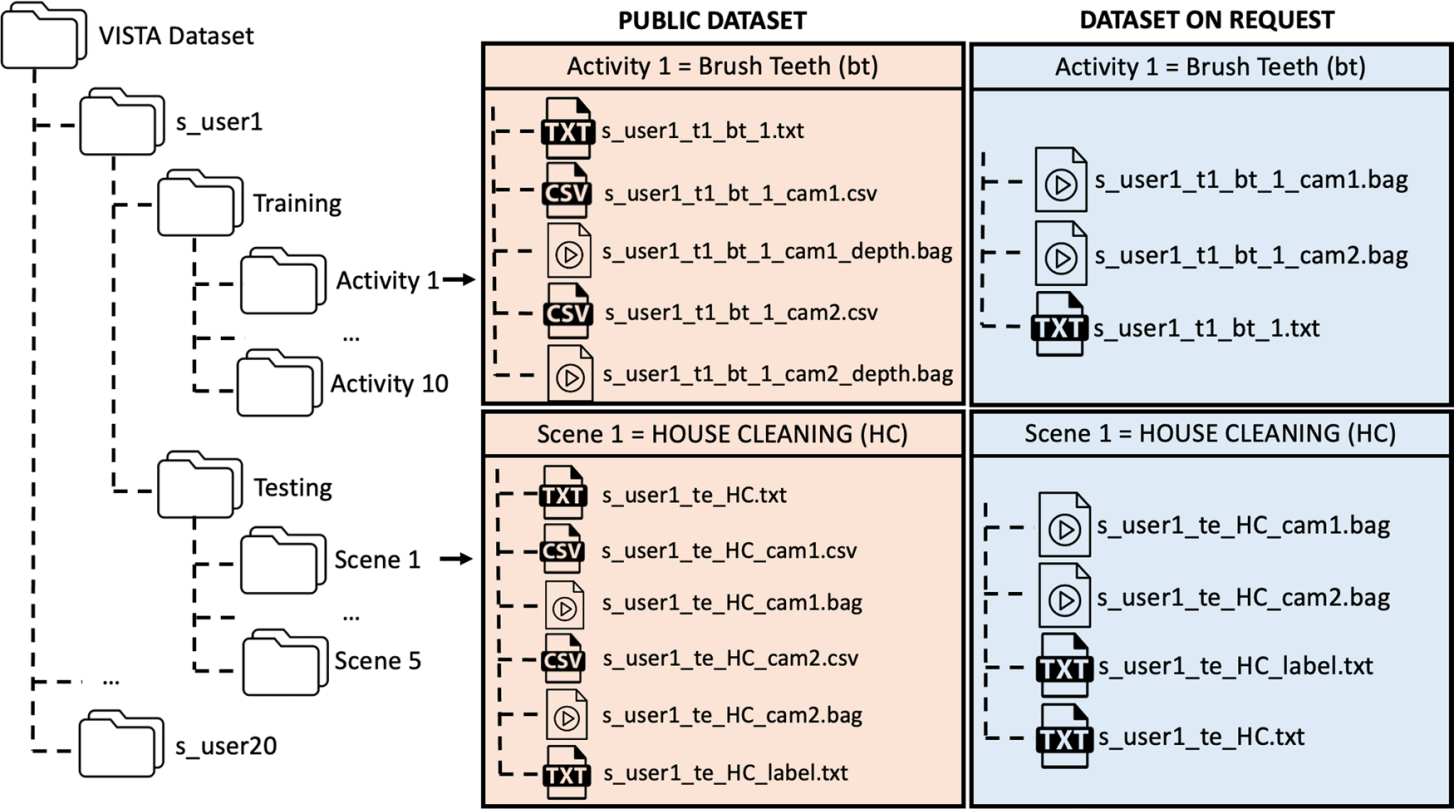
Organization of the VISTA database.

The organization of the two datasets is similar and is illustrated in Fig. 5. The names of the specific files depend on the specific activity that the subject performed during the experimentation. The files corresponding to each user are contained in a specific folder, whose name is the id of the user himself. Each folder, specific for each participant, contains two more folders: one with the data acquired during the phase 1 (i.e. Training) and the other containing the ones acquired during the phase 2 (i.e. Testing). In the former, there are ten more folders, one for each activity. In the latter, there are only five, one for each scene.

The *Public dataset* contains the following files for each activity or scene folder:

- a .txt file, which contains inertial data, as explained in more detail in the section “SensHand data organization”;
- two .csv files, which include the 2D skeleton coordinates extracted from the RGB images recorded by the two cameras, as detailed in the section “Camera data organization”;
- two .bag files, containing the depth images from the two cameras (see section “Camera data organization”);
- in the testing folder, a .txt label file containing information on the moments in time in which the person passed from one activity to another during the testing phase.

The *Dataset on request* is not anonymised, for this reason it will made available on request, exclusively for specific research purposes, under a Data Processing agreement. The files available in each action or scene folder of this dataset are:

- a .txt file, containing the inertial data (see section “SensHand data organization”);
- two .bag files, containing colored and depth images from the two cameras (see section “Camera data organization”);
- in the testing folder, a .txt label file containing information on the moments in time in which the person passed from one activity to another during the testing phase.

For both datasets, the string name of each file is made of:

- the id of the user: s_user1,.., s_user20;
- the phase: t1 for ‘Training’, te for ‘Testing’;
- the action, with the abbreviations reported in Table 2;
- the counter;
- only for the visual data, the indication of the camera: cam1 for the lateral camera, cam2 for the frontal one;
- only for the depth data, the depth label, indicating that the .bag files in the *Public dataset* contain only the depth information.

For instance, considering the *Public dataset*, while collecting data from s_user1, selecting the ‘Training 1’ and the action ‘brush teeth (BT)’, in case the “Start” button is pressed for the first time, the names of the files are:

- s_user1_t1_bt_1_cam1_depth.bag,
- s_user1_t1_bt_1_cam2_depth.bag,
- s_user1_t1_bt_1_cam1.csv,
- s_user1_t1_bt_1_cam2.csv
- s_user1_t1_bt_1.txt.
  Instead, by collecting data from s_user1, selecting ‘Testing’ phase and ‘HOUSE CLEANING (HC)’ scene, the names of the files are:
- s_user1_te_HC_cam1_depth.bag,
- s_user1_te_HC_cam2_depth.bag,
- s_user1_te_HC_cam1.csv
- s_user1_te_HC_cam2.csv
- s_user1_te_HC_label.txt,
- s_user1_te_HC.txt.

### SensHand data organization

The .txt file containing the inertial data has a table format, in which each package (each row) is characterized by the series of values reported below:

- Timestamp represents the record of the time of occurrence of the particular event, i.e. when a new packet with inertial data has arrived. This allows us to time the signal and synchronise it with the camera’s data. As far as rows are concerned, every time a new package is received, a line is added at the bottom of it.
- Count is an artificial counter which increments by 1 unit each time a new data packet is received and which resets when it reaches 255.
- CountMicro is the internal counter of the microcontroller which increments by 1 each time a new data packet is received and which resets when it reaches 255. If CountMicro corresponds to Count at each iteration, it means that the inertial glove SensHand didn’t lose any data.
- Freq is an artificial counter which increments by 1 unit each time a new data package is received and which resets when it reaches 100.
- Time is an artificial counter which increments by 1 each time Freq reaches 100. It keeps track of the time indicating how many seconds have passed, considering that the frequency of acquisition of the wearable glove is 100 Hz.
- Accx, Accy, Accz, Girx, Giry, Girz, Magx, Magy, Magz are the accelerometers, gyroscopes and magnetometers data along x,y,z axes for the wrist, thumb, index, and middle finger, respectively.

### Camera data organization

For what concerns the visual data, every time a new action or scene was performed, the data contained in the ROS messages were recorded in two appropriate files, one corresponding to the frontal camera and the other to the lateral one. The file containing the messages is called bag and has the extension.bag. The advantage offered by bag files is to have a recording that can be used several times, reproducing each time the exact operating scenario in which the bag was registered. The following list of topics are available for the bag files in the *Dataset on request*:

- /camera1/color/image_raw
- /camera2/color/image_raw
- /camera1/depth/image_rect_raw
- /camera2/depth/image_rect_raw

In order to reduce the dimensions of the bag files, we recorded a compressed version of the colored and depth images from the camera.

After the experimentation, the colored images were extracted from the bag files and were used to create video files. The OpenPose software was then employed to detect human body, hand, face and foot keypoints on single frames. For each video, the OpenPose output consisted in .json files containing the body part locations and detection confidence formatted as x_1,y_1,c_1,x_2,y_2,c_2, .., where *x* and *y* are the 2D coordinates of the skeleton, while *c* is the confidence score in the range [0, 1]. A total of 25 keypoints were estimated for the body; the coordinates are organized as showed in Table 4. The same approach has been followed to extract also 21 hand features, as follows:

- 0: wrist;
- from 1 to 4: joints of the thumb;
- from 5 to 8: joints of the index finger;
- from 9 to 12: joints of the middle finger;
- from 13 to 16: joints of the ring finger;
- from 17 to 20: joints of the little finger.

**Table 4:** Number of joint and associated joint type.

Number of joint	Joint type
0	Nose
1	Neck
2	Right Shoulder
3	Right Elbow
4	Right Wrist
5	Left Shoulder
6	Left Elbow
7	Left Wrist
8	Mid Hip
9	Right Hip
10	Right Knee
11	Right Ankle
12	Left Hip
13	Left Knee
14	Left Ankle
15	Right Eye
16	Left Eye
17	Right Ear
18	Left Ear
19	Left Big Toe
20	Left Small Toe
21	Left Heel
22	Right Big Toe
23	Right Small Toe
24	Right Heel
25	Background

All finger joints are numbered in ascending order from palm to fingertip. All the .json files were then converted to .csv files, which have been made available for the *Public dataset*. Scholars and researchers can select the most appropriate keypoints configuration to validate their algorithms. After extracting the skeleton information, the topics related to the colored images were manually eliminated from the bag files in the public version of the dataset, making it anonymous.

### Repository

The VISTA database, which comprosies both the *Public dataset* and *Dataset on request*, is available at this link 10.17032/shu-180021.16 To access any file on the repository, including the Readme file, there is a log in requirement. Interested people just register with their email address and a username and password. Within the supplementary material, we included a draft of the Data Processing Agreement for the access to the restricted *Dataset on request*. The *Public dataset* is available under the Creative Commons Attribution licence (CC-BY). Access to the *Dataset on request* is regulated by a Data Processing Agreement. For the *Public dataset*, licensees may copy, distribute, display and perform the work and make derivative works and remixes based on it only if they give the authors the full attribution, also including a citation to this article and the dataset on SHURDA^16^. The *Dataset on request* contains the raw RGB video data that could be made available on request under a Data Processing Agreement, which will only allow verification and validation of the research presented in this article.

## Technical Validation

In this section we provide a baseline analysis for the recognition of human activities, reporting some preliminary results of a possible application of the VISTA *Public dataset*. In particular, in this section, we will neglect the depth information obtained by the cameras and the magnetometer’s data retrieved by the SensHand. For this analysis, we merged and mixed the training and the testing parts of the *VISTA dataset* into one; this choice was made because training and testing data were acquired differently, but the samples of both were labeled with the id of the ten actions. As a first step, the raw data coming from the cameras and from SensHand were pre-processed, some features were extracted from them and then selected. This approach was carried out in parallel for the two sensor modalities, as described below.

### Pre-processing and feature extraction

**Fig. 6:**
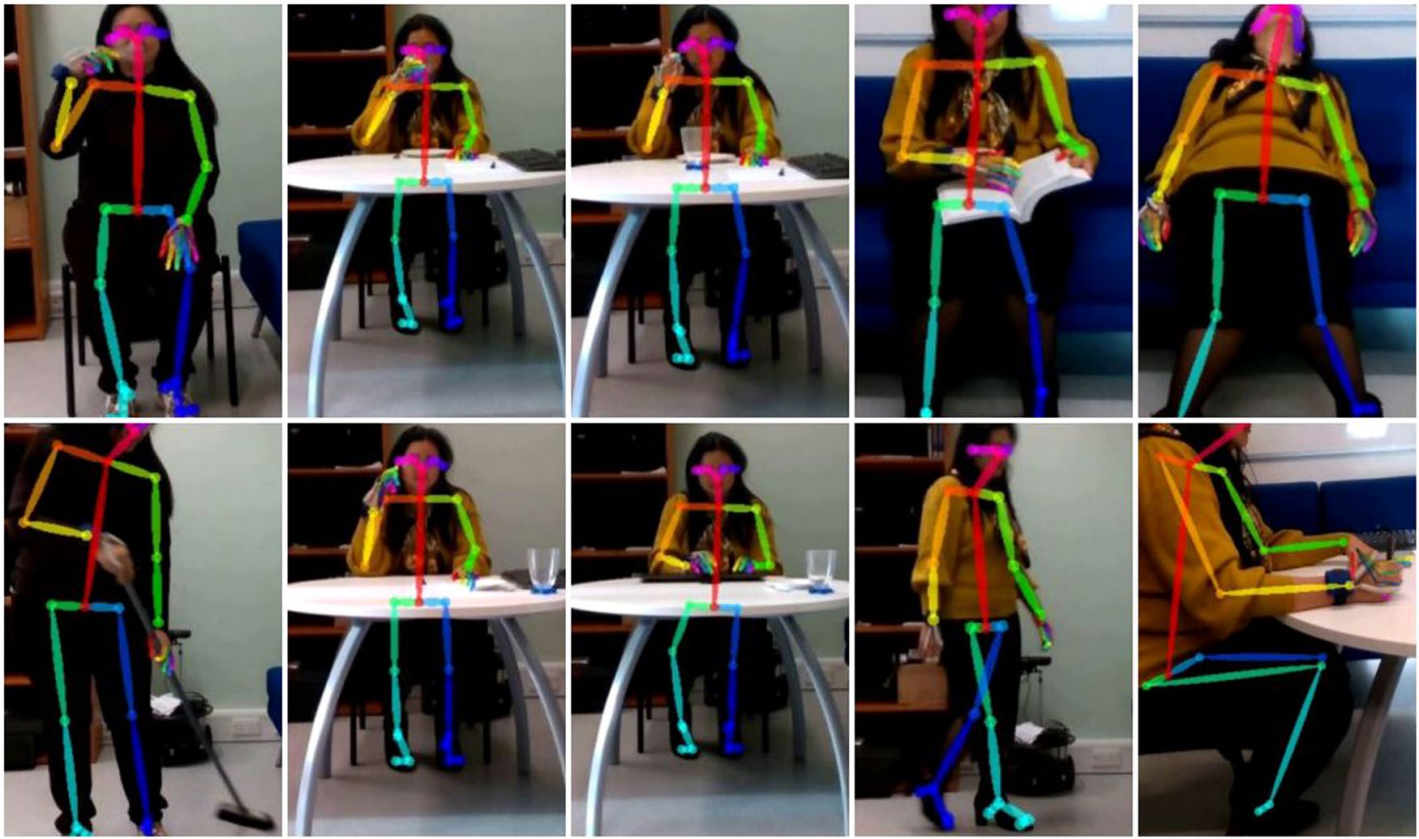
Skeleton tracking.

For what concerns the pre-processing and the extraction of the features related to the visual data, we followed the approach described in the Usage Notes to extract each frame from the bag files and to create the videos from the frames. Then, the OpenPose software was employed to retrieve the 2D coordinates of the skeleton’s joints, which constituted the features of the visual data, as showed in Fig. 6.

Based on the results obtained by previous analyses^17^, we only considered a subset of skeleton joints, such as head, neck, hands, feet and torso. This restricted set of joints correspond to the most significant joints of the body and has shown itself to be the most discriminating one for activity recognition, allowing reduction of complexity for the further steps of computation. The coordinates were then normalized considering the coordinates of the torso joint as a reference^17^, obtaining a final dataset which was independent from the person’s size and from the relative position of the camera. The final feature vector was composed by 12 attributes for each frame (x and y coordinates of head, neck, hands and feet). The signal was then segmented by 50%-overlapping moving windows with 3 seconds’ size, considering as final value of each window the mean value of each instance, i.e. each of the 12 attributes. This procedure was followed for the visual data acquired by both frontal and lateral camera. The size of the 3-second window was chosen taking into account the duration of each movement linked to the ten activities, which on average falls within 3 seconds.

Regarding the pre-processing and the extraction of the features related to the inertial data, the raw acceleration values from accelerometers and the raw angular velocity values from the gyroscopes were filtered. In particular, a 4th order digital low-pass Butterworth filter was used with the cut-off frequency set to 5 Hz. In the same way as the visual data, these data were then segmented into 3 seconds overlapping windows and several features were extracted from each window: 10 related to acceleration values (mean, standard deviation, variance, mean absolute deviation (MAD), root mean square (RMS), skewness, kurtosis, signal magnitude area (SMA), normalized jerk and power) and 6 to angular velocity ones (mean value, standard deviation, variance, MAD, RMS and power).

### Activity dataset creation and feature selection

At this stage, we created 5 separated datasets by considering the inertial and the visual features alone (Frontal Camera, Lateral Camera, Index finger, Wrist and Index finger + Wrist). In order to understand the reason behind the creation of the different datasets, it is important to highlight two aspects:

- for what concerns the visual data, we did not create combined datasets of lateral and frontal visual data. Indeed, the robot is placed frontally or laterally with respect to the person and it cannot be in both positions at the same time;
- for what concerns the inertial data, according to previous works^18^, we only considered data coming from the wrist and index finger sensors of SensHand.

After the extraction of the features and the creation of the datasets, we performed a feature selection separately for visual and inertial data. In particular, we first applied a Kruskal Wallis test, which confirmed that the ten gestures were statistically different for all the features under investigation (p < 0.05). Then, we performed a correlation analysis, through which only the statistically uncorrelated attributes were selected, considering a correlation coefficient of <0.85. For what concerns the visual features, all of them were statistically considered significant and uncorrelated, so the selected visual features matched the extracted ones. On the contrary, only a subset of inertial features was selected, as reported in Table 5.

**Table 5:** The first two columns show the features selected after the correlation analysis from the combined Index + Wrist dataset, while the third one shows the ones selected from Index and Wrist dataset when analysed on their own.

Index + Wrist		Index/Wrist
Wrist acc. mean	Index acc. mean	Acc. mean
Wrist acc. stdev	Index acc. stdev	Acc. stdev
Wrist acc. RMS	Index acc. RMS	Acc. RMS
Wrist acc. skewness	Index acc. skewness	Acc. skewness
Wrist acc. kurtosis	Index acc. kurtosis	Acc. kurtosis
Wrist acc. SMA	Index acc. SMA	Acc. SMA
Wrist acc. power	Index acc. power	Acc. power
Wrist ang. vel. mean	Index vel. power	Ang.vel. mean
Wrist ang. vel. power		Ang.vel. stdev
		Ang.vel. power

At this stage, we combined the inertial and the visual features through a fusion at feature-level, ending up with the eleven datasets listed below:

- FC: Frontal Camera
- LC: Lateral Camera
- I: Index finger
- W: Wrist
- IW: Index finger and Wrist
- I + FC: Index finger with Frontal Camera
- I + LC: Index finger with Lateral Camera
- W + FC: Wrist with Frontal Camera
- W + LC: Wrist with Lateral Camera
- IW + FC: Index finger and Wrist with Frontal Camera
- IW + LC: Index finger and Wrist with Lateral Camera

As previously stated, each signal was divided into overlapping windows. The number of rows in each dataset corresponded to the number of windows created. In total, 11043 windows were created for each combination of data concerning the frontal camera (FC, I + FC, W + FC, IW + FC) and inertial sensors alone (I, W, IW), while 10455 for the ones concerning the lateral camera (LC, I + LC, W + LC, IW + LC). As concerns the number of columns present in each dataset, it depended on the features retained after the feature selection process.

### Activity recognition

**Fig. 7:**
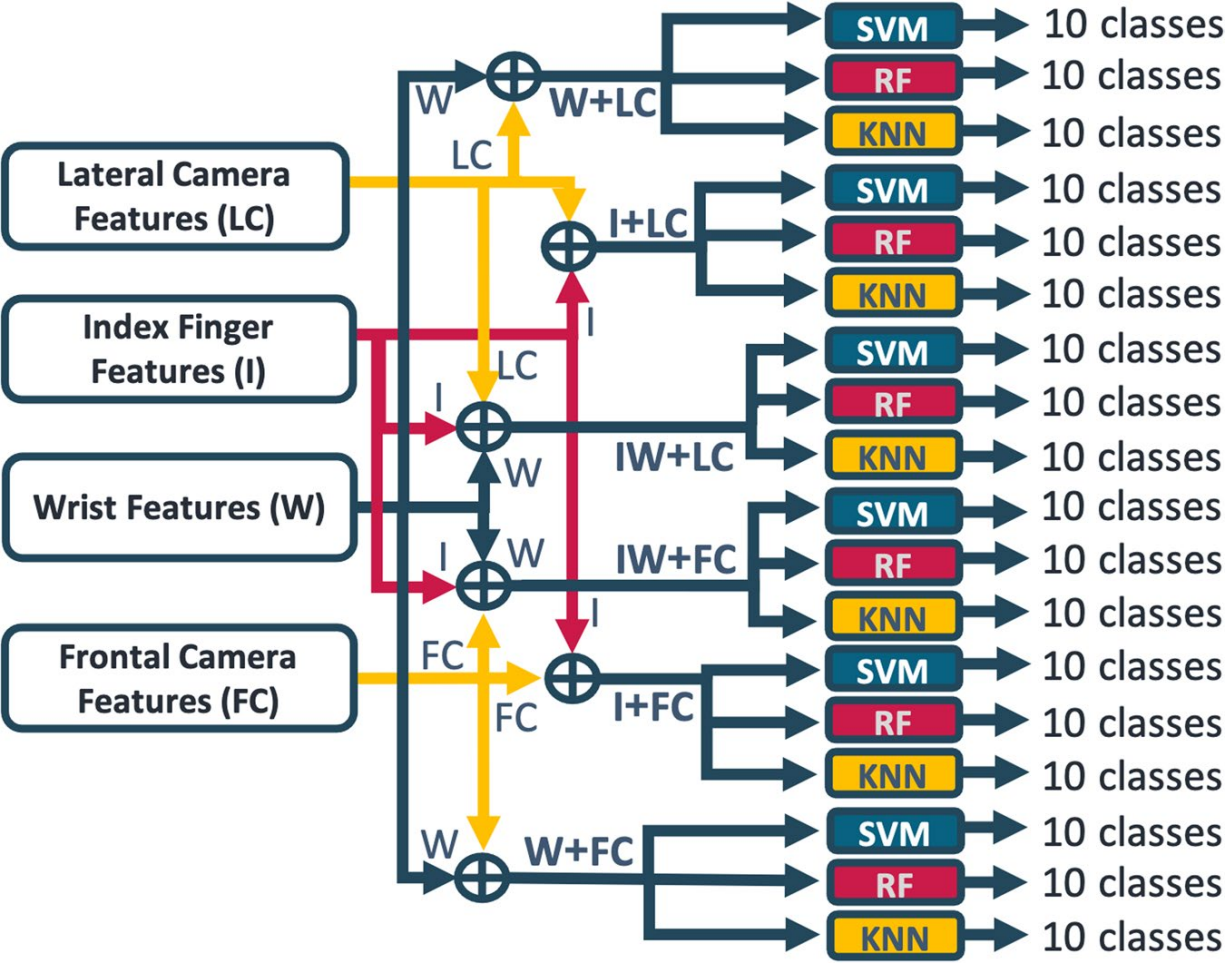
Scheme of the feature-level fusion.

After creating the datasets, we wanted to test them by performing human activity recognition, in particular making a distinction among the ten activities listed above. According to the state of the art, we chose three different supervised machine learning techniques, which are very often used to classify multi-modal data, such as inertial and visual ones: Support Vector Machine (SVM), Random Forest (RF) and K-Nearest Neighbor (KNN), as reported in Fig. 7.

The 11 datasets were classified through a 10-fold cross validation technique and the final results were obtained as the average on the performances of the ten models created after training the system. These datasets were analysed using Matlab2020a and the classification performances were evaluated in terms of accuracy, precision, f-measure and recall.

The results showed in Table 6 come from the stand-alone datasets, i.e. I, W, I + W, FC and LC. These evidences show that the system is able to recognize the ten gestures with 73% of accuracy, recall, f-measure and precision when considering the index or wrist sensors alone and 81% when combining the two of them. The camera alone performs better and allows the system to reach 90% of accuracy when it is frontally positioned and 81% when it is lateral.

**Table 6:** Results obtained by stand-alone systems.‘A’ stands for Accuracy, ‘R’ for Recall, ‘F’ for F-measure and ‘P’ for Precision.

	Index (I)				Wrist (W)				I + W			
	A	R	F	P	A	R	F	P	A	R	F	P
SVM	0.72	0.72	0.72	0.73	0.72	0.72	0.71	0.73	0.56	0.56	0.55	0.63
RF	0.56	0.57	0.57	0.59	0.58	0.57	0.57	0.60	0.66	0.66	0.66	0.67
KNN	0.73	0.73	0.73	0.73	0.73	0.73	0.73	0.73	0.81	0.81	0.81	0.82
		**Frontal camera (FC)**				**Lateral camera (LC)**						
		**A**	**R**	**F**	**P**	**A**	**R**	**F**	**P**			
	SVM	0.84	0.84	0.84	0.85	0.60	0.60	0.61	0.66			
	RF	0.71	0.72	0.72	0.72	0.64	0.65	0.64	0.65			
	KNN	0.90	0.90	0.90	0.90	0.81	0.81	0.82	0.81			

Similarly, we show the results after the fusion at feature-level in Table 7. In most cases, the combination of inertial sensors with visual ones enhance the capabilities of the system of recognizing activities of daily living. In particular, the system obtains 89% of accuracy with the combination of index sensor with the frontal camera and of index + wrist sensors with the frontal camera. 88% of accuracy is obtained by the combination of wrist sensor with the same camera. Instead, when considering the lateral camera, we obtain 77% of accuracy combining the camera with index or wrist sensors, and a big improvement is achieved by fusing index, wrist and lateral camera altogether.

**Table 7:** Fusion at Feature-level’s Results. ‘A’ stands for Accuracy, ‘R’ for Recall, ‘F’ for F-measure and ‘P’ for Precision.

	I + FC				W + FC				IW + FC			
	A	R	F	P	A	R	F	P	A	R	F	P
SVM	0.76	0.76	0.76	0.78	0.81	0.80	0.79	0.80	0.67	0.66	0.64	0.72
RF	0.75	0.75	0.76	0.77	0.76	0.77	0.77	0.77	0.77	0.78	0.78	0.79
KNN	0.89	0.89	0.89	0.89	0.88	0.88	0.89	0.88	0.89	0.89	0.89	0.90
	**I + LC**				**W + LC**				**IW + LC**			
	**A**	**R**	**F**	**P**	**A**	**R**	**F**	**P**	**A**	**R**	**F**	**P**
SVM	0.63	0.63	0.64	0.67	0.56	0.56	0.55	0.58	0.67	0.66	0.64	0.72
RF	0.74	0.75	0.75	0.76	0.73	0.73	0.73	0.74	0.77	0.78	0.78	0.79
KNN	0.77	0.77	0.78	0.82	0.77	0.77	0.79	0.82	0.89	0.89	0.89	0.90

**Fig. 8:**
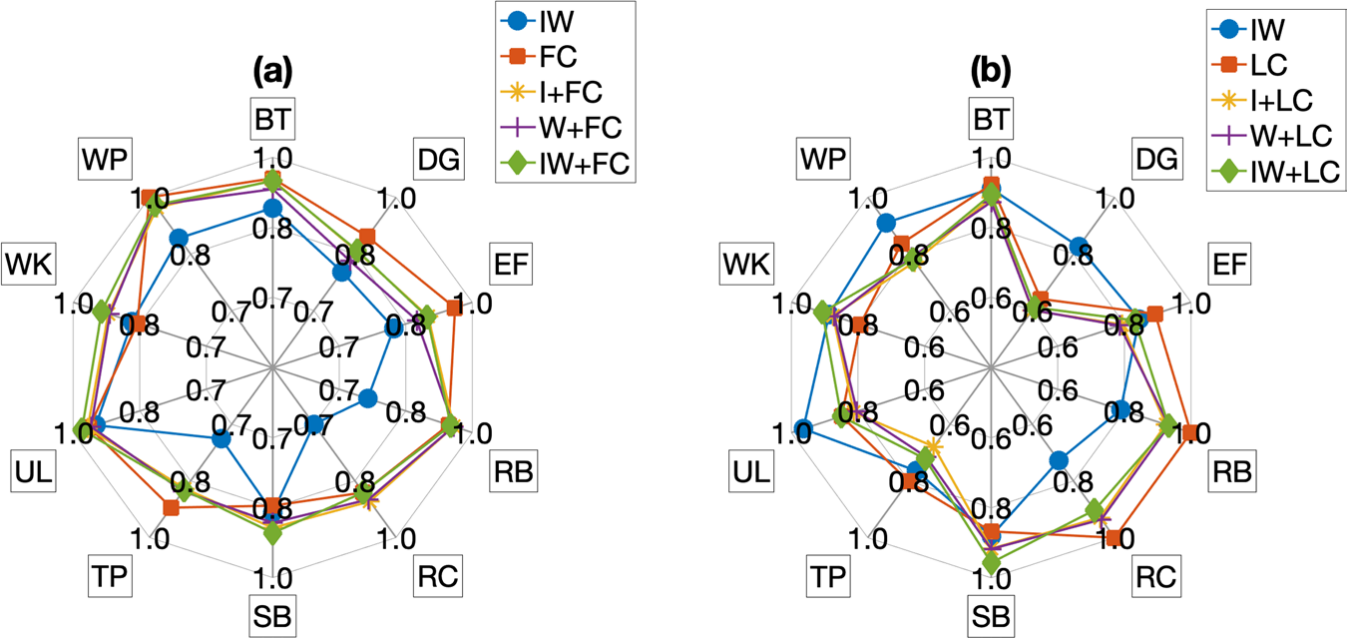
Spider plot which contains the F-measure values of the best classifiers on all datasets divided into those related to the frontal camera, on the left, and those related to the lateral one, on the right. All F-measure values are relative to individual actions.

In order to show the performances of the best classifiers on the single actions, in Fig. 8 we report a spider plot that shows the f-measure values of the best classifiers of all the combinations of datasets. This kind of analysis allowed us to study and compare different kinds of sensors to perform the best human action recognition. These evidences show that good results can be achieved with our dataset, reaching up to 90% of accuracy with the best configuration of sensors.

## Usage Notes

For the *Public dataset*, the steps needed to visualize the compressed depth bag files are the following ones:

1. Launch roscore
2. In a third terminal, to visualize the depth bag file, launch rosrun image_view image_view image: = /camera1/depth/image_rect_raw_image_transport: = compressedDepth
3. In a third terminal, enter the folder where the bag file is and launch rosbag play’namefile.bag’.

For what concerns the *Dataset on request*, the bag files contain also the colored information. Also in this case, the steps needed to visualize the compressed RGB bag files are similar:

1. Launch roscore
2. In a second terminal, to visualize the RGB bag file, launch rosrun image_view image_view image: = /camera1/color/image_raw _image_transport: = compressed
3. In a third terminal, enter the folder where the bag file is and launch rosbag play’namefile.bag’

Furthermore, the steps needed to convert rosbag files into videos are herein reported:

- Install ffmpeg in Ubuntu: sudo apt-get install ffmpeg
- To extract image frames from the rosbag file, create a folder where you will store images frames and move there. Enter the folder and execute the following command: rosrun image_view extract_images _sec_per_frame: = 0.01 image:=<IMAGETOPICINBAGFILE> where IMAGETOPICINBAGFILE is one of the topics listed by running rosbag info <BAGFILENAME>

- Open a new terminal and run rosbag play <BAGFILENAME>
- To obtain the video, in the images folder, run:ffmpeg –r <FPS> -b <BITRATE> -i frame%04d.jpg <OUTPUT>.avi where FPS is the number_of_messages_for_image_topic/duration_in_sec, BITRATE is KilyBytes/Sec and OUTPUT is the name of the video.

## Supplementary information

Supplementary material

## Data Availability

The lightweight processing needed to visualize and to decompress the bag files is described in the previous section. Processing code for data analysis is available on the supplementary material.
