# Supplementary material for: The VISTA datasets, a combination of inertial sensors and depth cameras data for activity recognition
Source: Sci Data. 2022 May 18;9:218. doi: 10.1038/s41597-022-01324-3 (PMC9117293; doi:10.1038/s41597-022-01324-3)
Supplement: Supplementary file 1 — Supplementary material [file 41597_2022_1324_MOESM1_ESM.pdf]

The VISTA dataset, a combination of inertial sensors and depth cameras for activity recognition

Laura Fiorini, Federica G. Cornacchia Loizzo, Alessandra Sorrentino, Erika Rovini, Alessandro Di Nuovo and Filippo Cavallo

SUPPLEMENTARY MATERIAL

DATA PROCESSING AGREEMENT TEMPLATE 2
DATA ANALYSIS (Matlab code)..7
Inertial data 7
Visual data 9
Feature Extraction 12
Feature Selection 14

DATA PROCESSING AGREEMENT TEMPLATE

This Data Processing Agreement ("**Agreement**") forms part of the Contract forServices ("**Principal Agreement**") between

Cornacchia Loizzo, Federica
Di Nuovo, Alessandro
Fiorini, Laura
Cavallo, Filippo
Sorrentino, Alessandra
Rovini, Erika

(the "**Creators**") of the VISTA, Visual and Inertial Sensor for recogniTion of human Activities database.

and

(the "**Data Processor**")

(together s the "**Parties**")

WHEREAS

(A) The Creators acts as a Data Controller.

(B) The Creators consent to use personal data of the VISTA database to the Data Processor for research purposes.

(C) The Parties seek to implement a data processing agreement that complies with the requirements of the current legal framework in relation to data processing and with the Regulation (EU) 2016/679 of the EuropeanParliament and of the Council of 27 April 2016 on the protection of naturalpersons with regard to the processing of personal data and on the free movement of such data, and repealing Directive 95/46/EC (General Data Protection Regulation).

(D) The Parties wish to lay down their rights and obligations.

IT IS AGREED AS FOLLOWS:

1. Definitions and Interpretation

1.1 Unless otherwise defined herein, capitalized terms and expressions used in this Agreement shall have the following meaning:

1.1.1 "**Agreement**" means this Data Processing Agreement and all Schedules;

1.1.2 "**VISTA Personal Data**" means any Personal Data Processed by a Data Processor with the written consent of the Creators pursuant to or in connection with the Principal Agreement;

1.1.3 "**Data Processor**" means a researcher that has access to the VISTA Personal Data for research purposes.

1.1.4 "**Data Protection Laws**" means EU Data Protection Laws and, to the extent applicable, the data protection or privacy laws of any other country;

1.1.5 "**EEA**" means the European Economic Area;

1.1.6 "**EU Data Protection Laws**" means EU Directive 95/46/EC, as transposed into domestic legislation of each Member State and as amended, replaced or superseded from time to time, including by the GDPR and laws implementing or supplementingthe GDPR;

1.1.7 "**GDPR**" means EU General Data Protection Regulation 2016/679;

1.1.8 "**Data Transfer**" means:

1.1.8.1 a transfer of VISTA Personal Data from the Creators to a Processor; or

1.1.8.2 an onward transfer of VISTA Personal Data from a Processor to a Subcontracted Processor, or between two establishments of a Processor,

in each case, where such transfer would be prohibited by Data Protection Laws (or by the terms of data transfer agreements put in place to address the data transfer restrictions of Data Protection Laws);

1.1.9 "**Services**" means the ________________________ services the Creators provides.

1.1.10 "**Subprocessor**" means any person appointed by or on behalf of Processor to process Personal Data on behalf of the Creators in connection with the Agreement.

1.2 The terms, "**Commission**", "**Controller**", "**Data Subject**", "**Member State**", "**Personal Data**", "**Personal Data Breach**", "**Processing**" and "**Supervisory Authority**" shall have the same meaning as in the GDPR, and their cognate terms shall be construed accordingly.

2. Processing of VISTA Personal Data

2.1 Processor shall:

2.1.1 comply with all applicable Data Protection Laws in the Processing of VISTA Personal Data; and

2.1.2 not Process VISTA Personal Data other than on the relevant Creators' documented instructions.

2.2 The Creators instructs Processor to process VISTA Personal Data.

3. Processor Personnel

Processor shall take reasonable steps to ensure the reliability of any employee, agent or contractor of any Contracted Processor who may haveaccess to the Creators Personal Data, ensuring in each case that access is strictly limited to those individuals who need to know / access the relevant Creators Personal Data, as strictly necessary for the purposes ofthe Principal Agreement, and to comply with Applicable Laws in the context of that individual's duties to the Contracted Processor, ensuring that all such individuals are subject to confidentiality undertakings or professional or statutory obligations of confidentiality.

4. Security

4.1 Taking into account the state of the art, the costs of implementation and the nature, scope, context and purposes of Processing as well as the risk of varying likelihood and severity for the rights and freedoms of natural persons, Processor shall in relation to the Creators Personal Data implement appropriate technical and organizational measures to ensure alevel of security appropriate to that risk, including, as appropriate, the measures referred to in Article 32(1) of the GDPR.

4.2 In assessing the appropriate level of security, Processor shall take accountin particular of the risks that are presented by Processing, in particular from a Personal Data Breach.

5. Subprocessing

5.1 Processor shall not appoint (or disclose any Creators Personal Data to) any Subprocessor unless required or authorized by the Creators.

6. Data Subject Rights

6.1 Taking into account the nature of the Processing, Processor shall assist the Creators by implementing appropriate technical and organizational measures, insofar as this is possible, for the fulfilment of the Creators obligations, as reasonably understood by Creators, to respond to requests to exercise Data Subject rights under the Data Protection Laws.

6.2 Processor shall:

6.2.1 promptly notify Creators if it receives a request from a Data Subject under any Data Protection Law in respect of Creators Personal Data; and

6.2.2 ensure that it does not respond to that request except on the documented instructions of Creators or as required by Applicable Laws to which the Processor is subject, in which case Processor shall to the extent permitted by Applicable Laws

inform Creators of that legal requirement before the ContractedProcessor responds to the request.

7. Personal Data Breach

7.1 Processor shall notify Creators without undue delay upon Processorbecoming aware of a Personal Data Breach affecting Creators Personal Data, providing Creators with sufficient information to allow the Creatorsto meet any obligations to report or inform Data Subjects of the Personal Data Breach under the Data Protection Laws.

7.2 Processor shall co-operate with the Creators and take reasonable commercial steps as are directed by Creators to assist in the investigation, mitigation and remediation of each such Personal Data Breach.

8. Data Protection Impact Assessment and Prior Consultation

Processor shall provide reasonable assistance to the Creators with any data protection impact assessments, and prior consultations with Supervising Authorities or other competent data privacy authorities, which Creators reasonably considers to be required by article 35 or 36 of the GDPR or equivalent provisions of any other Data Protection Law, in each case solely in relation to Processing of Creators Personal Data by, and taking into account the nature of the Processing and information available to, the Contracted Processors.

9. Deletion or return of Creators Personal Data

9.1 Subject to this section 9 Processor shall promptly and in any event within 10 business days of the date of cessation of any Services involving the Processing of Creators Personal Data (the "**Cessation Date**"), delete andprocure the deletion of all copies of those Creators Personal Data.

9.2 Processor shall provide written certification to Creators that it has fully complied with this section 9 within 10 business days of the Cessation Date.

10. Audit rights

10.1 Subject to this section 10, Processor shall make available to the Creators on request all information necessary to demonstrate compliance with thisAgreement, and shall allow for and contribute to audits, including inspections, by the Creators or an auditor mandated by the Creators in relation to the Processing of the Creators Personal Data by the Contracted Processors.

10.2 Information and audit rights of the Creators only arise under section 10.1to the extent that the Agreement does not otherwise give them information and audit rights meeting the relevant requirements of Data Protection Law.

11. Data Transfer

11.1 The Processor may not transfer or authorize the transfer of Data to countries outside the EU and/or the European Economic Area (EEA)without the prior written consent of the Creators. If personal dataprocessed under this Agreement is transferred from a country within the European Economic Area to a country outside the European Economic Area, the Parties shall ensure that the personal data are adequately protected. To achieve this, the Parties shall, unless agreed otherwise, rely on EU approved standard contractual clauses for the transfer of personal data.

12. General Terms

12.1 **Confidentiality.** Each Party must keep this Agreement and information itreceives about the other Party and its business in connection with this Agreement (“**Confidential Information**”) confidential and must not use or disclose that Confidential Information without the prior written consentof the other Party except to the extent that:

(a) disclosure is required by law;

(b) the relevant information is already in the public domain.

12.2 **Notices.** All notices and communications given under this Agreement must be in writing and will be delivered personally, sent by post or sent byemail to the address or email address set out in the heading of this Agreement at such other address as notified from time to time by the Parties changing address.

13. Governing Law and Jurisdiction

13.1 This Agreement is governed by the laws of the United Kingdom.

13.2 Any dispute arising in connection with this Agreement, which the Parties will not be able to resolve amicably, will be submitted to the exclusive jurisdiction of the courts of the United Kingdom.

IN WITNESS WHEREOF, this Agreement is entered into with effect from the datefirst set out below.

On behalf of the Creators

Signature____ Name:

____ Title:_

____ Date Signed:

____

Processor

Signature____ Name

____ Title_

____ Date Signed

____

DATA ANALYSIS (Matlab code)

Inertial data

```
%% Inertial data
% From the initial inertial dataset, I extracted only the data related to the
wrist and index finger.

wr_accx = data(:,5);
wr_accy = data(:,6);
wr_accz = data(:,7);
wr_gyrx = data(:,8);
wr_gyry = data(:,9);
wr_gyrz = data(:,10);
wr_magx = data(:,11);
wr_magy = data(:,12);
wr_magz = data(:,13);
in_accx = data(:,23);
in_accy = data(:,24);
in_accz = data(:,25);
in_gyrx = data(:,26);
in_gyry = data(:,27);
in_gyrz = data(:,28);
in_magx = data(:,29);
in_magy = data(:,30);
in_magz = data(:,31);

lastcolumn = data(:,end);

%% Fast Fourier Transform
% First, a Fourier analysis was performed to have a good idea of the frequencies
of the signal and the frequencies of the noise. In this case the main
frequencies of the signal were between 0 and 5, so a 4th order digital low-pass
Butterworth Filter was used to cut off all the other frequencies which only
represented noise. After that, the accelerations and angular velocities' norms
were computed.

fc = 5;
fs = 100;
[b,a] = butter(4, fc/(fs/2));
filteredwrist_accx = filter(b,a,wr_accx);
filteredwrist_accy = filter(b,a,wr_accy);
filteredwrist_accz = filter(b,a,wr_accz);
filteredindex_accx = filter(b,a,in_accx);
filteredindex_accy = filter(b,a,in_accy);
filteredindex_accz = filter(b,a,in_accz);

filteredwrist_avx = filter(b,a,wr_gyrx);
filteredwrist_avy = filter(b,a,wr_gyry);
filteredwrist_avz = filter(b,a,wr_gyrz);
filteredindex_avx = filter(b,a,in_gyrx);
filteredindex_avy = filter(b,a,in_gyry);
filteredindex_avz = filter(b,a,in_gyrz);

% Filtered Norm

% Accelerations
```

```
filteredwrist_acc = sqrt(filteredwrist_accx.^2 + filteredwrist_accy.^2 +
filteredwrist_accz.^2);
filteredindex_acc = sqrt(filteredindex_accx.^2 + filteredindex_accy.^2 +
filteredindex_accz.^2);

% Angular Velocities
filteredwrist_av = sqrt(filteredwrist_avx.^2 + filteredwrist_avy.^2 +
filteredwrist_avz.^2);
filteredindex_av = sqrt(filteredindex_avx.^2 + filteredindex_avy.^2 +
filteredindex_avz.^2);
```

Visual data

```
%% Visual data
% Starting from the csv file with the joints' coordinates extracted by OpenPose,
only some joints of interest were considered in the analysis.

Frames_cam1 = Skeleton_cam1(:,1);
Head_cam1 = Skeleton_cam1(:,2:4);
Neck_cam1 = Skeleton_cam1(:,5:7);
RHand_cam1 = Skeleton_cam1(:,14:16);
LHand_cam1 = Skeleton_cam1(:,23:25);
Torso_cam1 = Skeleton_cam1(:,26:28);
RFoot_cam1 = Skeleton_cam1(:,35:37);
LFoot_cam1 = Skeleton_cam1(:,44:46);
Labels_cam1 = Skeleton_cam1(:,end);

% STD NORM
dist2D_nt_cam1 = (sqrt((Neck_cam1(:,1)-Torso_cam1(:,1)).^2 + (Neck_cam1(:,2)-
Torso_cam1(:,2)).^2 ));
HeadSTD_cam1 = ((Head_cam1(:,1:2) - Torso_cam1(:,1:2)) ./ dist2D_nt_cam1);
NeckSTD_cam1 = ((Neck_cam1(:,1:2) - Torso_cam1(:,1:2)) ./ dist2D_nt_cam1);
LHandSTD_cam1 = ((LHand_cam1(:,1:2) - Torso_cam1(:,1:2)) ./ dist2D_nt_cam1);
RHandSTD_cam1 = ((RHand_cam1(:,1:2) - Torso_cam1(:,1:2)) ./ dist2D_nt_cam1);
LFootSTD_cam1 = ((LFoot_cam1(:,1:2) - Torso_cam1(:,1:2)) ./ dist2D_nt_cam1);
RFootSTD_cam1 = ((RFoot_cam1(:,1:2) - Torso_cam1(:,1:2)) ./ dist2D_nt_cam1);
jointsSTD_cam1 = [HeadSTD_cam1 NeckSTD_cam1 LHandSTD_cam1 RHandSTD_cam1
LFootSTD_cam1 RFootSTD_cam1 Labels_cam1];

%% Signal segmentation
% The signal was segmented by 3 seconds' windows with 50% overlapping.

% Inertial
n_window = 300;
overlap = 150;
n_start = 1;
n_max = length(filteredwrist_acc);
max_count = ceil((length(filteredwrist_acc)-n_window)/(n_window-overlap))+1;

for count = 1: max_count
    n_end = n_start + n_window - 1;
    if n_end > n_max
        wr_acc_segm(count, 1:n_max-n_start+1) =
filteredwrist_acc(n_start:n_max);
        wr_accx_segm(count, 1:n_max-n_start+1) =
filteredwrist_accx(n_start:n_max);
        wr_accy_segm(count, 1:n_max-n_start+1) =
filteredwrist_accy(n_start:n_max);
        wr_accz_segm(count, 1:n_max-n_start+1) =
filteredwrist_accz(n_start:n_max);
        wr_av_segm(count, 1:n_max-n_start+1) = filteredwrist_av(n_start:n_max);
        in_acc_segm(count, 1:n_max-n_start+1) =
filteredindex_acc(n_start:n_max);
        in_accx_segm(count, 1:n_max-n_start+1) =
filteredindex_accx(n_start:n_max);
        in_accy_segm(count, 1:n_max-n_start+1) =
filteredindex_accy(n_start:n_max);
        in_accz_segm(count, 1:n_max-n_start+1) =
filteredindex_accz(n_start:n_max);
```

```
        in_av_segm(count, 1:n_max-n_start+1) = filteredindex_av(n_start:n_max);
        label_segm(count, 1:n_max-n_start+1) = lastcolumn(n_start:n_max);
    else
        wr_acc_segm(count,:) = filteredwrist_acc(n_start:n_end);
        wr_accx_segm(count,:) = filteredwrist_accx(n_start:n_end);
        wr_accy_segm(count,:) = filteredwrist_accy(n_start:n_end);
        wr_accz_segm(count,:) = filteredwrist_accz(n_start:n_end);
        wr_av_segm(count,:) = filteredwrist_av(n_start:n_end);
        in_acc_segm(count,:) = filteredindex_acc(n_start:n_end);
        in_accx_segm(count,:) = filteredindex_accx(n_start:n_end);
        in_accy_segm(count,:) = filteredindex_accy(n_start:n_end);
        in_accz_segm(count,:) = filteredindex_accz(n_start:n_end);
        in_av_segm(count,:) = filteredindex_av(n_start:n_end);
        label_segm(count,:) = lastcolumn(n_start:n_end);
    end
    n_start = n_end - overlap;
end

% Cameras

n_window_cam = round(fps * 3);
overlap_cam = round(n_window_cam/2);
jointsSTD_n_start = 1;
jointsSTD_n_max = length(jointsSTD_cam1);
jointsSTD_max_count = ceil((length(jointsSTD_cam1)-n_window_cam)/(n_window_cam-
overlap_cam))+1;

for jointsSTD_count= 1: jointsSTD_max_count
    jointsSTD_n_end = jointsSTD_n_start + n_window_cam - 1;
    if jointsSTD_n_end > jointsSTD_n_max
        jointsSTD_1_cam1(jointsSTD_count, 1:jointsSTD_n_max-jointsSTD_n_start+1)
= jointsSTD_cam1(jointsSTD_n_start:jointsSTD_n_max,1);
        jointsSTD_2_cam1(jointsSTD_count, 1:jointsSTD_n_max-jointsSTD_n_start+1)
= jointsSTD_cam1(jointsSTD_n_start:jointsSTD_n_max,2);
        jointsSTD_3_cam1(jointsSTD_count, 1:jointsSTD_n_max-jointsSTD_n_start+1)
= jointsSTD_cam1(jointsSTD_n_start:jointsSTD_n_max,3);
        jointsSTD_4_cam1(jointsSTD_count, 1:jointsSTD_n_max-jointsSTD_n_start+1)
= jointsSTD_cam1(jointsSTD_n_start:jointsSTD_n_max,4);
        jointsSTD_5_cam1(jointsSTD_count, 1:jointsSTD_n_max-jointsSTD_n_start+1)
= jointsSTD_cam1(jointsSTD_n_start:jointsSTD_n_max,5);
        jointsSTD_6_cam1(jointsSTD_count, 1:jointsSTD_n_max-jointsSTD_n_start+1)
= jointsSTD_cam1(jointsSTD_n_start:jointsSTD_n_max,6);
        jointsSTD_7_cam1(jointsSTD_count, 1:jointsSTD_n_max-jointsSTD_n_start+1)
= jointsSTD_cam1(jointsSTD_n_start:jointsSTD_n_max,7);
        jointsSTD_8_cam1(jointsSTD_count, 1:jointsSTD_n_max-jointsSTD_n_start+1)
= jointsSTD_cam1(jointsSTD_n_start:jointsSTD_n_max,8);
        jointsSTD_9_cam1(jointsSTD_count, 1:jointsSTD_n_max-jointsSTD_n_start+1)
= jointsSTD_cam1(jointsSTD_n_start:jointsSTD_n_max,9);
        jointsSTD_10_cam1(jointsSTD_count, 1:jointsSTD_n_max-
jointsSTD_n_start+1) = jointsSTD_cam1(jointsSTD_n_start:jointsSTD_n_max,10);
        jointsSTD_11_cam1(jointsSTD_count, 1:jointsSTD_n_max-
jointsSTD_n_start+1) = jointsSTD_cam1(jointsSTD_n_start:jointsSTD_n_max,11);
        jointsSTD_12_cam1(jointsSTD_count, 1:jointsSTD_n_max-
jointsSTD_n_start+1) = jointsSTD_cam1(jointsSTD_n_start:jointsSTD_n_max,12);
        label_cam1(jointsSTD_count, 1:jointsSTD_n_max-jointsSTD_n_start+1) =
jointsSTD_cam1(jointsSTD_n_start:jointsSTD_n_max,13);
    else
```

```
        jointsSTD_1_cam1(jointsSTD_count,:) =
jointsSTD_cam1(jointsSTD_n_start:jointsSTD_n_end,1);
        jointsSTD_2_cam1(jointsSTD_count,:) =
jointsSTD_cam1(jointsSTD_n_start:jointsSTD_n_end,2);
        jointsSTD_3_cam1(jointsSTD_count,:) =
jointsSTD_cam1(jointsSTD_n_start:jointsSTD_n_end,3);
        jointsSTD_4_cam1(jointsSTD_count,:) =
jointsSTD_cam1(jointsSTD_n_start:jointsSTD_n_end,4);
        jointsSTD_5_cam1(jointsSTD_count,:) =
jointsSTD_cam1(jointsSTD_n_start:jointsSTD_n_end,5);
        jointsSTD_6_cam1(jointsSTD_count,:) =
jointsSTD_cam1(jointsSTD_n_start:jointsSTD_n_end,6);
        jointsSTD_7_cam1(jointsSTD_count,:) =
jointsSTD_cam1(jointsSTD_n_start:jointsSTD_n_end,7);
        jointsSTD_8_cam1(jointsSTD_count,:) =
jointsSTD_cam1(jointsSTD_n_start:jointsSTD_n_end,8);
        jointsSTD_9_cam1(jointsSTD_count,:) =
jointsSTD_cam1(jointsSTD_n_start:jointsSTD_n_end,9);
        jointsSTD_10_cam1(jointsSTD_count,:) =
jointsSTD_cam1(jointsSTD_n_start:jointsSTD_n_end,10);
        jointsSTD_11_cam1(jointsSTD_count,:) =
jointsSTD_cam1(jointsSTD_n_start:jointsSTD_n_end,11);
        jointsSTD_12_cam1(jointsSTD_count,:) =
jointsSTD_cam1(jointsSTD_n_start:jointsSTD_n_end,12);
        label_cam1(jointsSTD_count,:) =
jointsSTD_cam1(jointsSTD_n_start:jointsSTD_n_end,13);
    end
    jointsSTD_n_start = jointsSTD_n_end - overlap_cam;
end
```

Feature Extraction

```
% For each window, different features were extracted from inertial data: mean,
standard deviation, variance, mean absolute deviation (MAD), root mean square
(RMS), skewness, kurtosis, signal magnitude area (SMA), normalized jerk and
power.

% Wrist
% Accelerations
wr_mean_acc = mean(wr_acc_segm,2);
wr_stdev_acc = std(wr_acc_segm,0,2);
wr_var_acc = var(wr_acc_segm,0,2);
wr_mad_acc = mad(wr_acc_segm,0,2);
wr_rms_acc = rms(wr_acc_segm,2);
wr_skewness_acc = skewness(wr_acc_segm,1,2);
wr_kurtosis_acc = kurtosis(wr_acc_segm,1,2);
wr_SMA_acc = sum(abs(wr_accx_segm),2) + sum(abs(wr_accy_segm),2) +
sum(abs(wr_accz_segm),2);
wr_jerk_diff_acc = diff(wr_acc_segm,1,2)./(1/fs);
wr_jerk_mean_acc = mean(wr_jerk_diff_acc,2);
wr_rmse_JERK_acc = sqrt(1/length(wr_jerk_diff_acc).*sum((wr_jerk_diff_acc-
wr_jerk_mean_acc).^2,2));
wr_FFT_acc_segm = fft(wr_acc_segm);
wr_pow_acc = wr_FFT_acc_segm.*conj(wr_FFT_acc_segm);
wr_pow_acc = sum(wr_pow_acc,2);

% Angular Velocities
wr_mean_av = mean(wr_av_segm,2);
wr_stdev_av = std(wr_av_segm,0,2);
wr_var_av = var(wr_av_segm,0,2);
wr_mad_av = mad(wr_av_segm,0,2);
wr_rms_av = rms(wr_av_segm,2);
wr_FFT_av_segm = fft(wr_av_segm);
wr_pow_av = wr_FFT_av_segm.*conj(wr_FFT_av_segm);
wr_pow_av = sum(wr_pow_av,2);

wr_features= [wr_mean_acc wr_stdev_acc wr_var_acc wr_mad_acc wr_rms_acc
wr_skewness_acc wr_kurtosis_acc wr_SMA_acc wr_rmse_JERK_acc wr_pow_acc
wr_mean_av wr_stdev_av wr_var_av wr_mad_av wr_rms_av wr_pow_av];

% Index
% Accelerations
in_mean_acc = mean(in_acc_segm,2);
in_stdev_acc = std(in_acc_segm,0,2);
in_var_acc = var(in_acc_segm,0,2);
in_mad_acc = mad(in_acc_segm,0,2);
in_rms_acc = rms(in_acc_segm,2);
in_skewness_acc = skewness(in_acc_segm,1,2);
in_kurtosis_acc = kurtosis(in_acc_segm,1,2);
in_SMA_acc = sum(abs(in_accx_segm),2) + sum(abs(in_accy_segm),2) +
sum(abs(in_accz_segm),2);
in_jerk_diff_acc = diff(in_acc_segm,1,2)./(1/fs);
in_jerk_mean_acc = mean(in_jerk_diff_acc,2);
in_rmse_JERK_acc = sqrt(1/length(in_jerk_diff_acc).*sum((in_jerk_diff_acc-
in_jerk_mean_acc).^2,2));
in_FFT_acc_segm = fft(in_acc_segm);
```

```
in_pow_acc = in_FFT_acc_segm.*conj(in_FFT_acc_segm);
in_pow_acc = sum(in_pow_acc,2

% Angular Velocities
in_mean_av = mean(in_av_segm,2);
in_stdev_av = std(in_av_segm,0,2);
in_var_av = var(in_av_segm,0,2);
in_mad_av = mad(in_av_segm,0,2);
in_rms_av = rms(in_av_segm,2);
in_FFT_av_segm = fft(in_av_segm);
in_pow_av = in_FFT_av_segm.*conj(in_FFT_av_segm);
in_pow_av = sum(in_pow_av,2);

in_features= [in_mean_acc in_stdev_acc in_var_acc in_mad_acc in_rms_acc
in_skewness_acc in_kurtosis_acc in_SMA_acc in_rmse_JERK_acc in_pow_acc
in_mean_av in_stdev_av in_var_av in_mad_av in_rms_av in_pow_av];

% For what concerns the cameras, the mean values of the joints’ coordinates were
computed for each window.
jointsSTD_1_cam1 = mean(jointsSTD_1_cam1,2);
jointsSTD_2_cam1 = mean(jointsSTD_2_cam1,2);
jointsSTD_3_cam1 = mean(jointsSTD_3_cam1,2);
jointsSTD_4_cam1 = mean(jointsSTD_4_cam1,2);
jointsSTD_5_cam1 = mean(jointsSTD_5_cam1,2);
jointsSTD_6_cam1 = mean(jointsSTD_6_cam1,2);
jointsSTD_7_cam1 = mean(jointsSTD_7_cam1,2);
jointsSTD_8_cam1 = mean(jointsSTD_8_cam1,2);
jointsSTD_9_cam1 = mean(jointsSTD_9_cam1,2);
jointsSTD_10_cam1 = mean(jointsSTD_10_cam1,2);
jointsSTD_11_cam1 = mean(jointsSTD_11_cam1,2);
jointsSTD_12_cam1 = mean(jointsSTD_12_cam1,2);
label_cam1 = mode(label_cam1,2);

joints_cam1 = [jointsSTD_1_cam1 jointsSTD_2_cam1 jointsSTD_3_cam1
jointsSTD_4_cam1 jointsSTD_5_cam1 jointsSTD_6_cam1 jointsSTD_7_cam1
jointsSTD_8_cam1 jointsSTD_9_cam1 jointsSTD_10_cam1 jointsSTD_11_cam1
jointsSTD_12_cam1];
```

Feature Selection

```
% Kruskal Wallis
kw = zeros(size(data_features,2),1);

 for i=1:(size(data_features,2)-1)
     kw(i) = kruskalwallis(data_features(:,i),data_features(:,end),'off');
 end

parameters = find(kw < 0.05);
matrix = data_features(:,parameters);

% Finally, the correlated features were removed (correlation coefficient <
0.85).
columns= CorrelationAnalysis(matrix,0.85);
FinalDataset = matrix(:,columns');
```
